# Characterization of vaginal microbiota diversity by 16S rRNA high-throughput sequencing

**DOI:** 10.3389/fmicb.2026.1777216

**Published:** 2026-04-20

**Authors:** Menglu Chen, Jinling Li, Hetao Chen, Adilai Tuerxun, Mengfen Mao, Jiahui Song, Boya Li, Yaonan Mo, Xiandun Zhai

**Affiliations:** 1 Luoyang Key Laboratory of Zebrafish Toxicology Research, Luoyang Key Laboratory of Transplantation and Immunological Studies for Haematological Diseases, Department of Clinical Laboratory, the First Affiliated Hospital, College of Clinical Medicine of Henan University of Science and Technology, Luoyang, China; 2 School of Basic Medicine and Forensic Medicine, Henan University of Science and Technology, Luoyang, China; 3 Criminal Investigation Brigade of Xuanwu Sub-bureau, Nanjing Public Security Bureau, Nanjing, China

**Keywords:** 16S rRNA, forensic genetics, high-throughput sequencing, individual identification, vaginal microbiota

## Abstract

**Introduction:**

This study employed high-throughput sequencing to analyze the diversity of vaginal microbiota in healthy women of reproductive age, thereby establishing a foundational dataset that may inform future forensic applications such as individual identification.

**Methods:**

A cohort of 100 healthy reproductive-age women meeting the inclusion criteria was enrolled. Vaginal secretion samples were collected, after which microbial DNA was extracted and assessed for quality using agarose gel electrophoresis. The V3-V4 region of the 16S rRNA gene was subsequently amplified with universal primers and subjected to Illumina high-throughput sequencing for comprehensive microbial analysis.

**Results:**

Sequencing analysis revealed that the five most predominant genera in the female vaginal microbiota were *Lactobacillus*, *Gardnerella*, *Prevotella*, *Serratia*, and *Atopobium*. Cluster analysis of the 100 samples classified them into five major clusters, further delineated into eight subtypes, ranging from absolute dominance of *Lactobacillus* to dominance of non-*Lactobacillus* bacteria. Analysis of influence factors showed that microbial diversity was positively associated with age, higher in urban compared to suburban participants, and lower in pregnant versus non-pregnant women. The 16S rRNA high-throughput sequencing of vaginal microbiota exhibited distinct inter-group clustering, with *Lactobacillus* identified as a core candidate marker for vaginal secretions.

**Discussion:**

The compositional profile of the vaginal microbiota exhibited variations associated with individual characteristics such as age range and geographic region. This study provides a foundational description of vaginal microbiota composition in this cohort. Such foundational data are a necessary first step toward exploring potential future applications in forensic science, though direct inference of individual characteristics is not supported by the current dataset.

## 1 Introduction

Microorganisms are ubiquitous and play essential roles in ecological processes. The Human Microbiome Project (HMP) (Turnbaugh et al., 2007) has revealed that the human body hosts an immense microbial community, often referred to as the “second genome” (Grice and Segre, 2012). This microbiota is pervasive, highly diverse, and exhibits inter-individual variability.

Microbial DNA can retain information about tissue origin, leveraging a complementary tool in forensic science (Allwood et al., 2020; Giampaoli et al., 2018). For instance, previous studies have demonstrated the forensic utility of microbiota with skin microbiome achieving up to 94% accuracy in host identification, and bite mark microbiota showing high concordance with perpetrators (García et al., 2020; Schmedes et al., 2018). Beyond individual identification, microorganisms can function as candidate markers for specific bodily fluids, such as, *Streptococcus* for saliva, and *Lactobacillus* for vaginal secretions (Grice and Segre, 2012). Furthermore, Microbial evidence can assist in estimating the time and cause of death, as well as clarifying key details of the crime scene (Allwood et al., 2020; Metcalf et al., 2017; Schmedes et al., 2020).

The vaginal microbiota, typically dominated by *Lactobacillus* species, exhibits lower diversity compared to oral and gut microbiomes in healthy adult women (Liang et al., 2022; Chen et al., 2021; France et al., 2022; Auriemma et al., 2021). Its relatively enclosed environment renders it less susceptible to external perturbations than skin or oral microbiota. It exhibits distinct group-specific and individual spatiotemporal characteristics due to long-term stabilizing factors such as ethnicity, genetics, age, menstrual cycle, geographic region, and behavioral pattern (Allwood et al., 2020; Shuting and Rongxia, 2018; Huang et al., 2019; Yao et al., 2021). Consequently, vaginal microbiota holds promising potential for forensic applications.

With the advancement of high-throughput sequencing (Bing-Bing, 2017; Børsting and Morling, 2015), 16S rRNA gene sequencing has emerged as a cost-effective and widely adopted approach for microbial community profiling, offering sufficient resolution for large-scale comparative studies (Sa et al., 2017).

Therefore, the main objective of this study was to employ 16S rRNA gene high-throughput sequencing to characterize the composition and community types of the vaginal microbiota in healthy reproductive-age women, as well as its variations across different age groups, pregnancy statuses, and geographical regions. The results are expected to provide foundational data on vaginal microbiota composition, offering a scientific basis for future explorations into its potential forensic applications.

## 2 Materials and methods

### 2.1 Sample source

Vaginal swab samples were collected from women attending routine gynecological examinations at the First Affiliated Hospital of Henan University of Science and Technology between October 2022 and March 2023. A total of 100 volunteers aged 20–50 years were recruited and assigned unique identifiers (S1–S100) in chronological order of enrollment. The samples primarily documented information on age, pregnancy status, region, marital status, and vaginal status (Sample details are provided in Supplementary Table S1). After collection, samples were temporarily stored at 4 °C–8 °C and transferred within 24 h to a −20 °C freezer for long-term preservation.

Inclusion Criteria: Eligible participants were reproductive-aged women who met all of the following criteria: (1) regular menstrual cycles and no use of antibiotics or antifungals for at least 1 month prior to sampling; (2) no vaginal interventions (e.g., douching or topical medication) within 48 h before sample collection; (3) negative microscopic examination for pus cells, *Candida*, *Trichomonas*, and *Mycoplasma*, a vaginal pH ≤ 4.6, and no clinical diagnosis of bacterial vaginosis; (4) absence of vaginal bleeding at the time of sampling; and (5) no history of systemic diseases such as diabetes mellitus or autoimmune disorders.

The study protocol and informed consent procedures were reviewed and approved by the Ethics Committee of the First Affiliated Hospital of Henan University of Science and Technology (No. 2023-600).

### 2.2 Sample pre-processing

The vaginal swab was thawed in a sterile tube and replenished with double-distilled water until the tip was fully submerged. After rinsing and pressing well, the tip was discarded. The resulting bacterial solution was transferred to a 1.5 mL EP tube and centrifuged to facilitate bacterial settling. The excess supernatant was carefully removed, retaining a volume of 250 μL. To ensure resuspension of the bacteria, the bacterial solution was vigorously vortexed.

### 2.3 Total DNA extraction and concentration and purity testing

The total DNA of vaginal secretion microbial genomes was extracted using the E. Z.N. A. Universal Pathogen DNA Kit D4035 (Omega Bio-Tek, USA). The extraction procedure strictly followed the manufacturer’s instructions, with ultrapure water utilized as a negative quality control. The concentration and purity of the extracted DNA were determined using a FLA6000 UV microspectrophotometer (Hangzhou Ltd.). The extracted DNA was subsequently stored at −20 °C for further downstream processes.

### 2.4 PCR amplification and sequencing

The V3-V4 region of the 16S rRNA gene was amplified using the universal primers 338F (forward) and 806R (reverse), which were synthesized by Biotech Bioengineering (Shanghai) Co. The primer sequences were as follows: Forward: 5′-ACTCCTACGGGAGGCAGCA-3′, Reverse: 5′-GGACTACCAGGGTATCTAAT-3′. The amplified fragment had a length of 468 bp. PCR amplification was conducted using the A100-Thermal Cycler (Hangzhou Longji Scientific Instruments Co., Ltd.). The reaction mixture consisted of 2 μL of 10 × PCR Buffer (containing Mg^2+^), 1.6 μL of 2.5 mM dNTPs, 0.2 μL of 10 μM forward and reverse primers each, 0.2 μL of 5 U/μL TaKaRa Ex-Taq enzyme, 1 μL of template DNA, and ultrapure water was added to bring the final volume to 20 μL. The cycling parameters were as follows: initial denaturation at 98 °C for 2 min, followed by 30 cycles of denaturation at 95 °C for 30 s, annealing at 56 °C for 30 s, extension at 72 °C for 30 s, and a final extension at 72 °C for 10 min. Quality control was conducted using ultrapure water and human peripheral venous blood DNA. The PCR products were visualized by 2.0% agarose gel electrophoresis. Finally, the qualified products were subjected to Illumina Miseq PE300 double-end high-throughput sequencing analysis performed by Biotech Bioengineering (Shanghai) Co., Ltd.

### 2.5 Data processing and statistical analysis

#### 2.5.1 Data processing analysis

Raw sequencing data were demultiplexed and quality-filtered using QIIME2 (v2020.11). Primers were removed from the 5′ end of reads using cutadapt (v4.0). Quality filtering was performed with the DADA2 plugin in QIIME2. Reads were truncated at 280 bp (forward) and 220 bp (reverse) based on the visualization of quality profiles; reads with ambiguous bases (N) or an expected error >2 were discarded. After quality control, samples with read depths below 10,000 were excluded from downstream analysis.

High-quality sequences were clustered into operational taxonomic units (OTUs) at 97% similarity threshold using the UPARSE algorithm implemented in USEARCH (v11.0.667). The clustering procedure followed these steps: (1) extracting non-replicate sequences from each sample to reduce computational redundancy; (2) merging dereplicated sequences across all samples and removing singleton sequences; (3) clustering non-replicate sequences (excluding singletons) at 97% similarity, with chimera removal during clustering to obtain OTU representative sequences; (4) mapping all quality-filtered sequences to OTU representative sequences at ≥97% similarity to generate the OTU abundance table. For taxonomic assignment, representative sequences of the OTUs were compared against the microbial reference database RDP 16S rRNA database (RDP Release 11, http://rdp.cme.msu.edu/) using the RDP classifier (v2.12) with a minimum confidence threshold of 0.8. Taxonomic assignments were made from domain to genus level; species-level assignments were considered tentative due to the limited resolution of 16S rRNA gene fragments.

Alpha diversity indices, including the Chao1 index (estimating richness), ACE index (abundance-based coverage estimator), Simpson index (diversity), and Shannon index (richness and evenness), were calculated using Mothur software (v 3.8.31) after rarefying all samples to the minimum sequencing depth (10,000 reads per sample) to account for uneven sequencing effort.

Beta diversity analysis was performed to assess microbial community dissimilarities between samples. Principal Coordinate Analysis (PCoA) was conducted based on Bray–Curtis dissimilarity matrices using the vegan package (v2.5-6) in R (v4.0.3). Permutational multivariate analysis of variance (PERMANOVA) with 999 permutations was used to test for significant differences in microbial community composition between groups.

Linear Discriminant Analysis Effect Size (LEfSe) (v 1.1.0) was used to identify taxa with significant abundance differences between groups. The threshold for logarithmic LDA score was set to 2.0, and the significance level for the Kruskal–Wallis test was *p* < 0.05. All visualizations were generated using R software (v4.0.3).

#### 2.5.2 Statistical analysis

All statistical analyses were conducted using SPSS software (version 16.0). For continuous measurement data, the Shapiro–Wilk test and Levene’s test were first applied to assess normality and homogeneity of variances, respectively, to determine the appropriate use of parametric or non-parametric tests. Categorical data were analyzed using the Chi-square test or Fisher’s exact test, and ordinal data were analyzed using non-parametric rank-sum tests.

For comparisons between two groups, if the data met the assumptions of normality and homogeneity of variances, an independent samples t-test was used; otherwise, the Mann–Whitney U test was applied. For comparisons among three or more groups, if the data satisfied the assumptions of normality and homogeneity of variances, one-way analysis of variance (ANOVA) was performed. If a significant overall difference was detected, post-hoc pairwise comparisons were conducted using the LSD method. If the data violated the assumptions of normality or homogeneity of variances, the Kruskal–Wallis H test was used. Following a significant result, pairwise comparisons were performed using Dunn’s test.

The initial significance level (*α*) for all hypothesis tests was set at 0.05. For all analyses involving multiple comparisons, *p*-values were adjusted using the False Discovery Rate (FDR) correction. Only FDR-corrected *p*-values are reported in the text and figures. A corrected *p*-value of less than 0.05 was considered statistically significant.

## 3 Results

### 3.1 Demographic distribution analysis

In this study, the 100 enrolled samples were grouped according to age, pregnancy status, and region (Table 1), and a difference analysis was conducted on their population distribution. Significant differences were observed in the distribution among different age groups (*p* < 0.05), while no significant differences were found in the distribution based on pregnancy status or region.

**Table 1:** Distribution of volunteers by age, pregnancy status, and region.

Characteristic	Group	Category	Number
Age group	A	20–29 years	34
	B	30–39 years	51
	C	40–50 years	15
Pregnancy status	P	Pregnancy	52
	N	Non-pregnancy	48
Region		Urban	54
		Suburban	46

### 3.2 Electrophoresis results of PCR products

**Figure 1:**
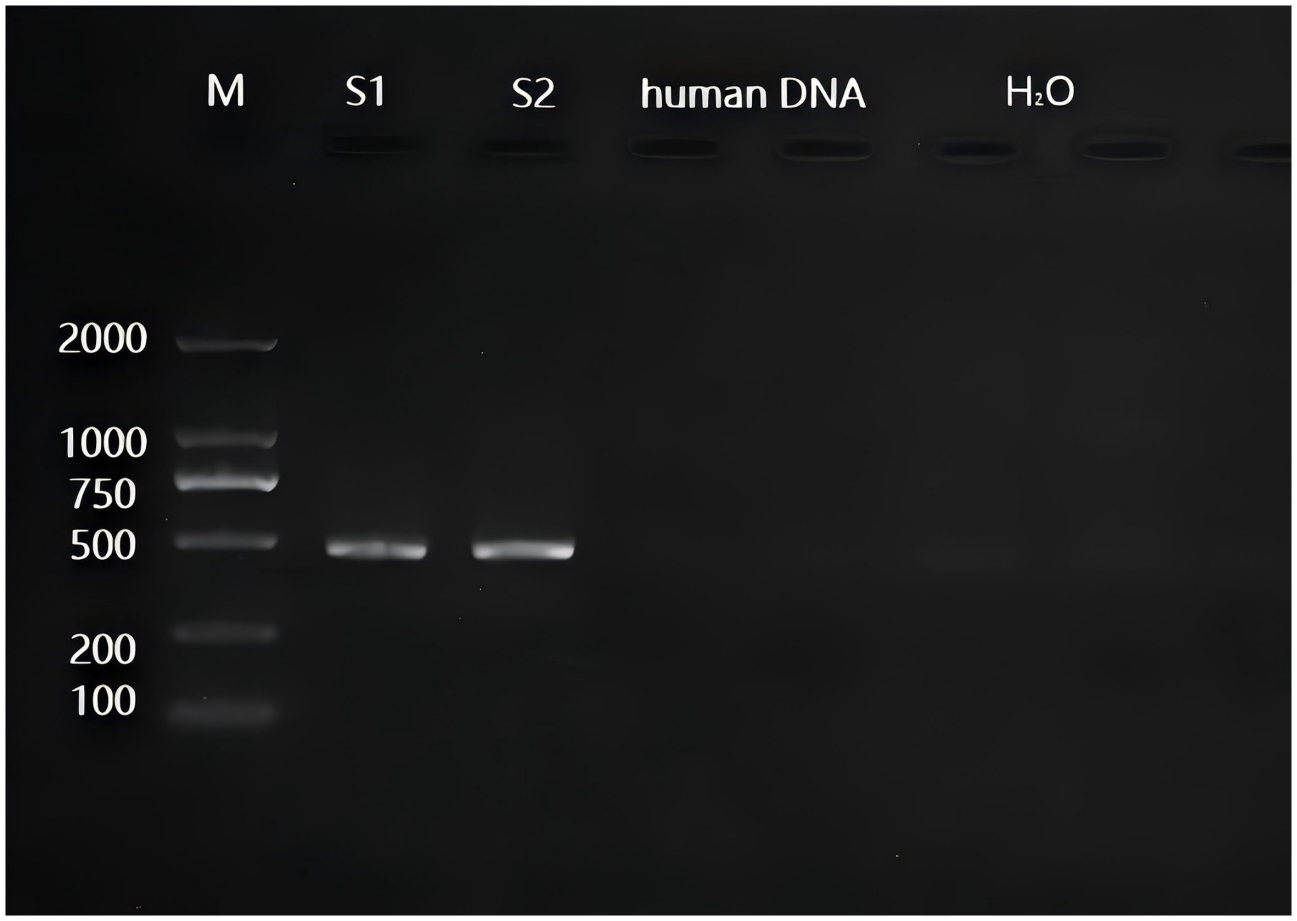
Electrophoresis diagram of amplification products of some samples. Note: M indicates the Marker; S1, S2 indicates sample 1 and sample 2; Human DNA, H_2_O indicates quality control; “Human DNA” and “H_2_O” are both duplicates of two lanes.

The total extracted DNA was quantified using an ultraviolet spectrophotometer, and the A260/A280 ratio ranged from 1.7 to 2.0, indicating good purity of the DNA samples. The average concentration of the samples was approximately 23.23 ng/μL. Agarose gel electrophoresis was performed to visualize the PCR products, as shown in Figure 1. The amplified bands appeared as clear and bright bands without any trailing.

### 3.3 Sequencing and quality control

**Figure 2:**
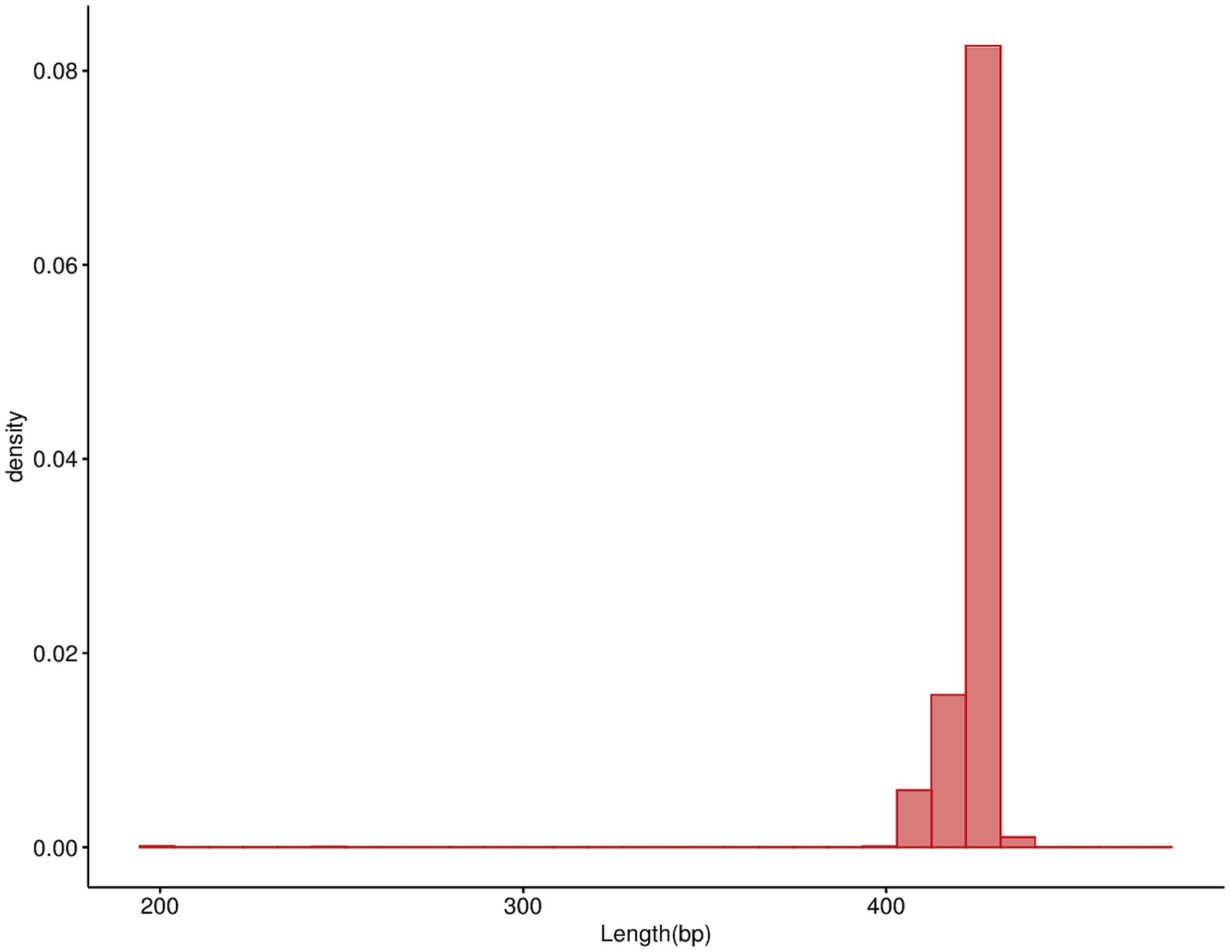
Distribution of effective sequence data length.

Illumina sequencing was conducted on 100 samples, resulting in a total of 16922791 valid sequences after filtering out low-quality sequences, splicing, and removing chimeras. On average, each sample yielded approximately 169227.91 sequences. The length distribution of the valid sequences is illustrated in Figure 2, showing that the length of the valid sequences in all samples fell within the range of 400–500 bp. This consistency suggests efficient and specific amplification.

### 3.4 Structural composition of the vaginal microbiota in the total sample of reproductive-age women

**Figure 3:**
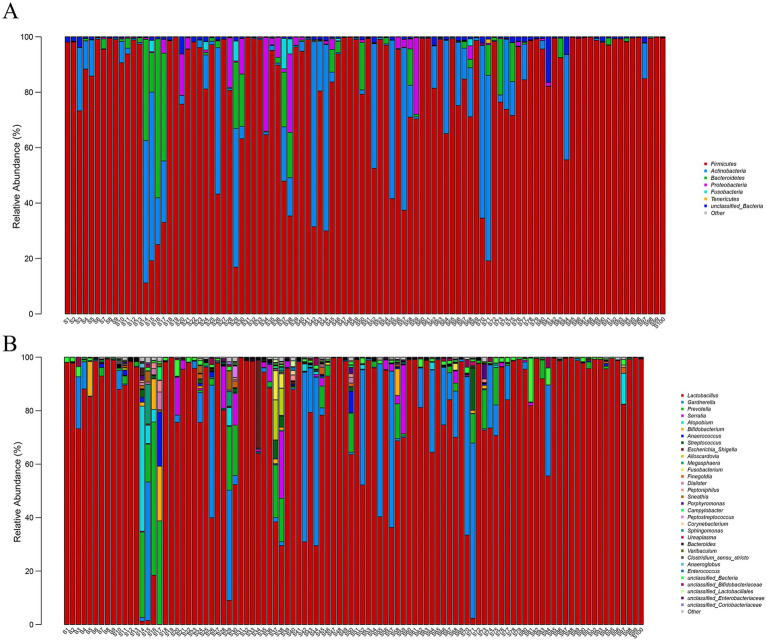
Relative abundance of the total microbiota community. **(A)** Phylum levels; **(B)** Genus levels.

A total of 1,490 OTUs were identified through annotated statistical analysis of the sequencing results. The community structure of the samples was visualized at the phylum and genus levels, and a stacked bar chart of the relative abundance of species was generated. Species with an abundance of less than 1% were grouped under “others.” At the phylum level, *Firmicutes* was the most dominant phylum, followed by *Actinobacteria*, *Bacteroidetes*, *Proteobacteria*, and *Fusobacteria* (Figure 3A). Corresponding to the genus level (Figure 3B), the largest dominant genus was *Lactobacillus* under *Firmicutes*, with a proportion of more than 90%, followed by *Gardnerella*, *Prevotella*, *Serratia*, *Atopobium*, *Bifidobacterium*, *Anaerococcus*, etc. Notably, a small proportion of samples presented a distinct profile, characterized by the absence of *Lactobacillus* dominance and the presence of diverse non-*Lactobacillus* polymicrobial communities.

**Figure 4:**
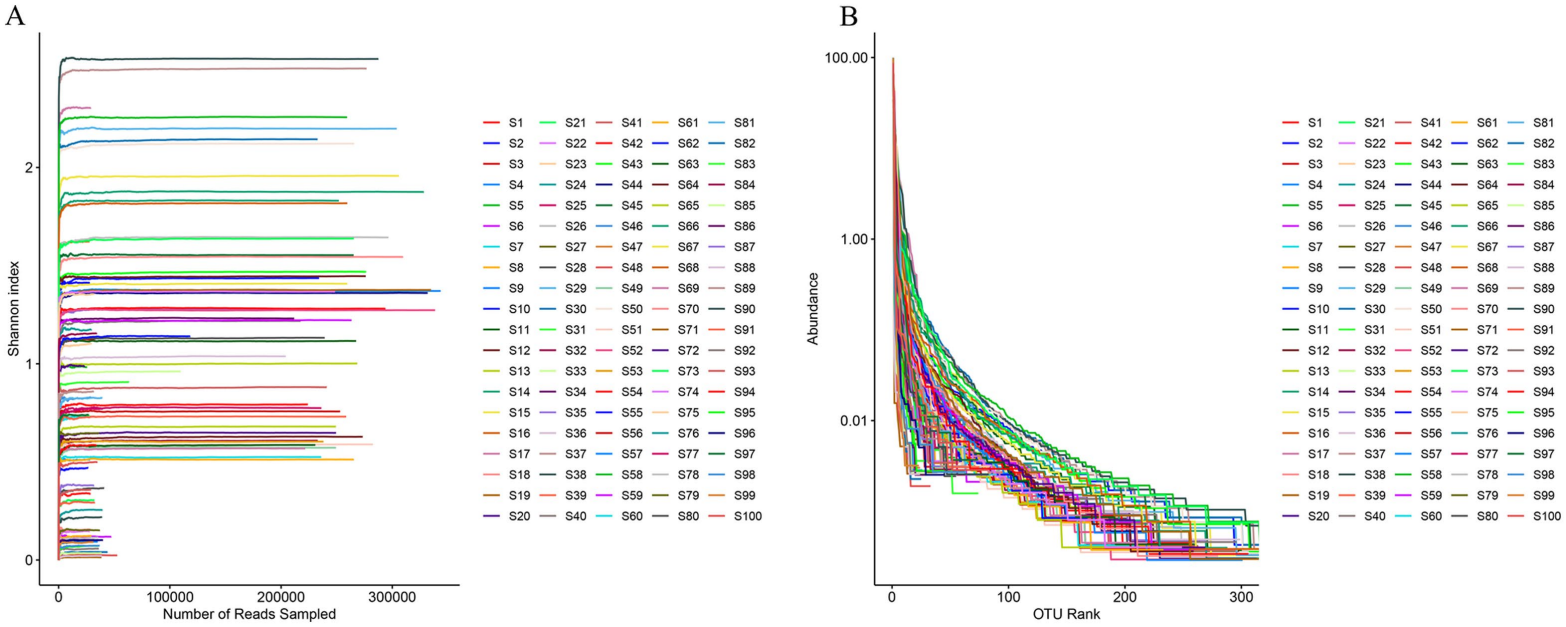
Assessment of sample sequencing depth curves. **(A)** Rank-abundance curves; **(B)** rarefaction curves.

The Rank-abundance curve (Figure 4A) was generated using the relative abundance of OTUs as the vertical coordinate and the rank of OTUs as the horizontal coordinate. This curve reflects the richness and evenness of species within the microbial community. As the rank of OTUs increased, the curve exhibited a wider and flatter shape, indicating a diverse and evenly distributed species composition in the samples. To assess the adequacy of sequencing depth, a certain number of sequences were randomly selected from the sequencing data, and the number of OTUs they represented was determined to construct the OTUs rarefaction curve (Figure 4B). As the number of extracted sequences increased, the rarefaction curves of all samples gradually plateaued, indicating that the sequencing volume for these samples was sufficient and the sequencing depth met the required qualifications. These results suggest that the sample’s sequencing data was representative and suitable for subsequent analyses.

**Figure 5:**
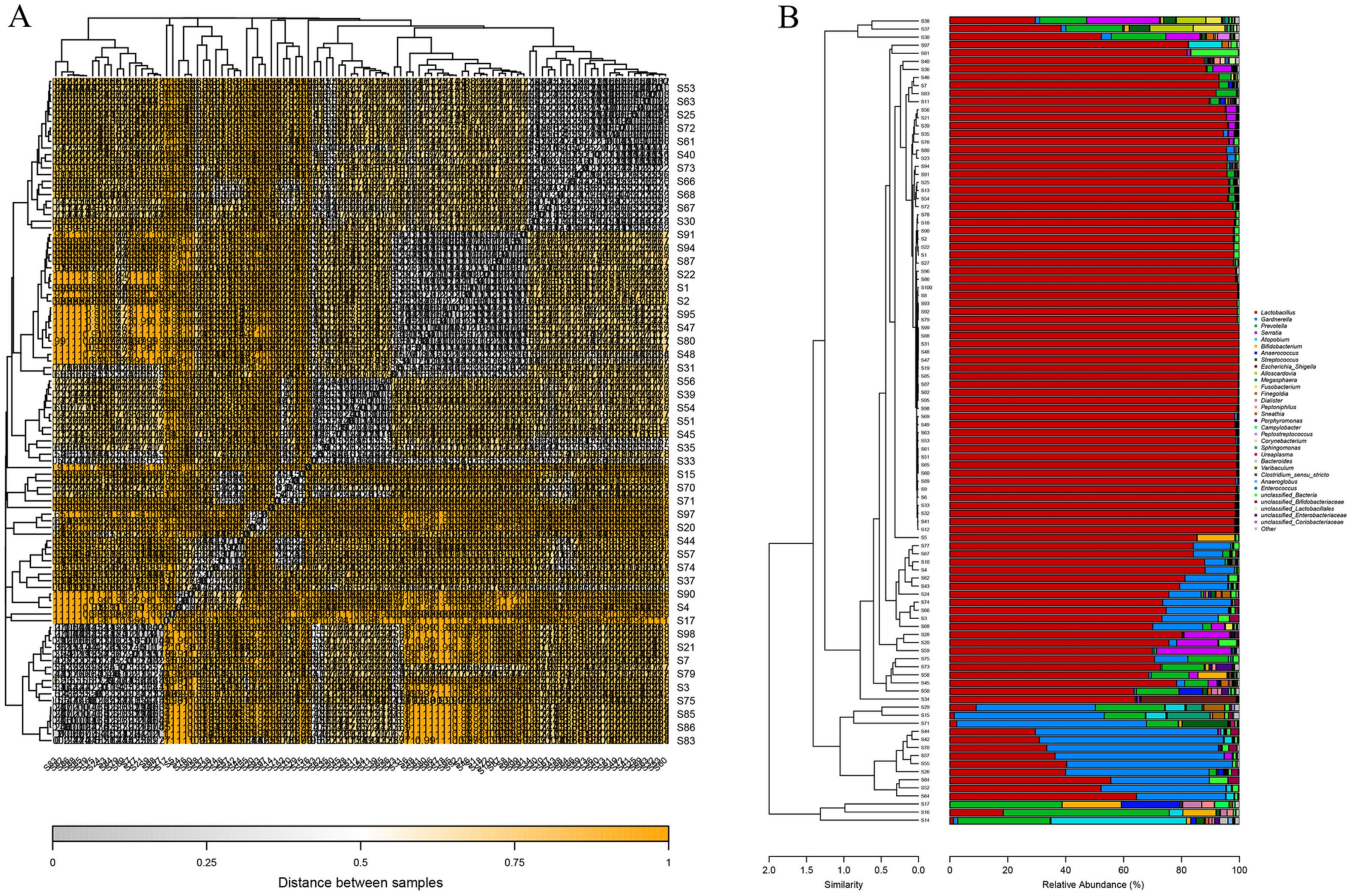
Clustering of all samples. **(A)** Distance heatmap; **(B)** clustering tree with genus-level composition bar plots.

The samples were subjected to further clustering analysis. Sample correlation analysis was utilized to construct a distance heat map (Figure 5A), and cluster analysis based on sample abundance was performed to generate a tree bar plot (Figure 5B). The 100 sequenced samples were grouped into five major clusters and eight subtypes. At the genus level, the clusters were as follows: the first cluster consisted of samples where *Lactobacillus* spp. was the predominant organism; the second cluster comprised samples with *Lactobacillus* as the dominant organism, accompanied by the presence of *Gardnerella, Prevotella, and Serratia*, resulting in three subtypes; the third cluster showed a balanced presence of *Lactobacillus* and *Gardnerella*; the fourth cluster exhibited a smaller dominance of *Lactobacillus* along with *Prevotella* and *Serratia*; and the fifth cluster comprised samples characterized by the presence of *non-Lactobacillus* bacteria, specifically *Gardnerella* and *Prevotella*, resulting in two subtypes.

### 3.5 Analysis of diversity and comparison of differences under different groupings

#### 3.5.1 Analysis of vaginal microbial diversity in reproductive-age women from different age groups

**Figure 6:**
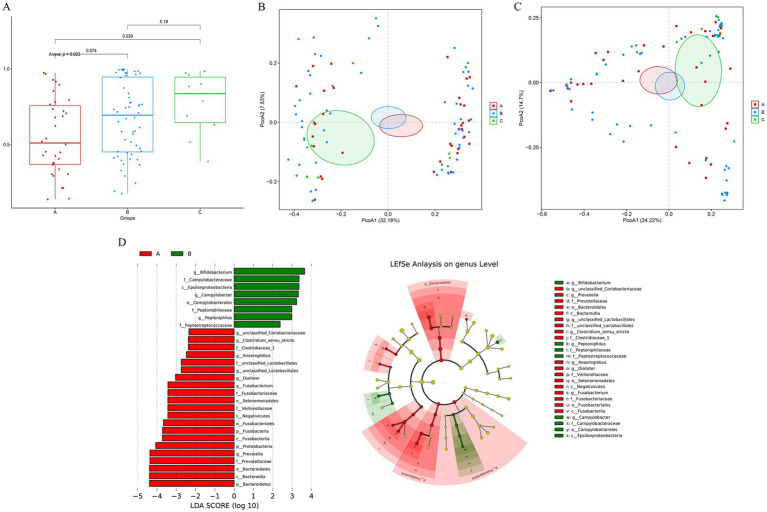
Microbial diversity and composition across age groups. **(A)** Box plot of the Shannon index; **(B)** unweighted and **(C)** weighted PCoA plots; **(D)** LEfSe analysis identifying differentially abundant taxa among groups. Note: colored nodes represent taxa with significant discriminatory power; yellow nodes indicate non-significant taxa.

To investigate age-related differences in the vaginal microbiota of reproductive-age women, analyses were conducted on three age groups. Microbial abundance and diversity were assessed using alpha and beta diversity analyses. Alpha diversity, measured by the Shannon index, showed an increasing trend with age (Figure 6A), suggesting that vaginal microbiota diversity rises with advancing age. However, only the difference between Group A and Group C remained statistically significant after FDR correction (corrected *p =* 0.039). This may be because Group A represents women at peak reproductive age, whereas Group C is approaching menopause, leading to a more pronounced disparity in their vaginal microbiota. In contrast, the smaller age gaps and relatively stable physiological states between Groups A/B and B/C likely account for the lack of significant differences in microbial diversity.

UniFrac distance based unweighted and weighted PCoA revealed distinct clustering of the three age groups (Figures 6B,C). Groups A and B exhibited closer proximity and overlapping, while being clearly separated from Group C. This indicates that Group C demonstrates greater dissimilarity compared to Groups A and B. To identify species differences among the groups and explore potential candidate marker genera that may vary between groups, we performed LEfSe analysis, which calculates Linear Discriminant Analysis (LDA) values and generates corresponding evolutionary trees to represent the effect size of species abundance differences (Figure 6D). In this study, a threshold of 2.0 was applied to the Linear Discriminant Analysis (LDA) scores to identify differentially abundant taxa, where a higher LDA score signifies a greater effect size. Notable differences were found between groups, with *Bifidobacterium* spp., *Aspergillus* spp., and *Campylobacter* spp. being significantly more abundant in Group A than in Groups B and C. Conversely, Group B showed a significantly higher abundance of *Bacteroidetes*, *Bacteroides*, and *Prevotella* spp. compared to both Group A and Group C.

#### 3.5.2 Analysis of vaginal microbial diversity in women during pregnancy and non-pregnancy

**Figure 7:**
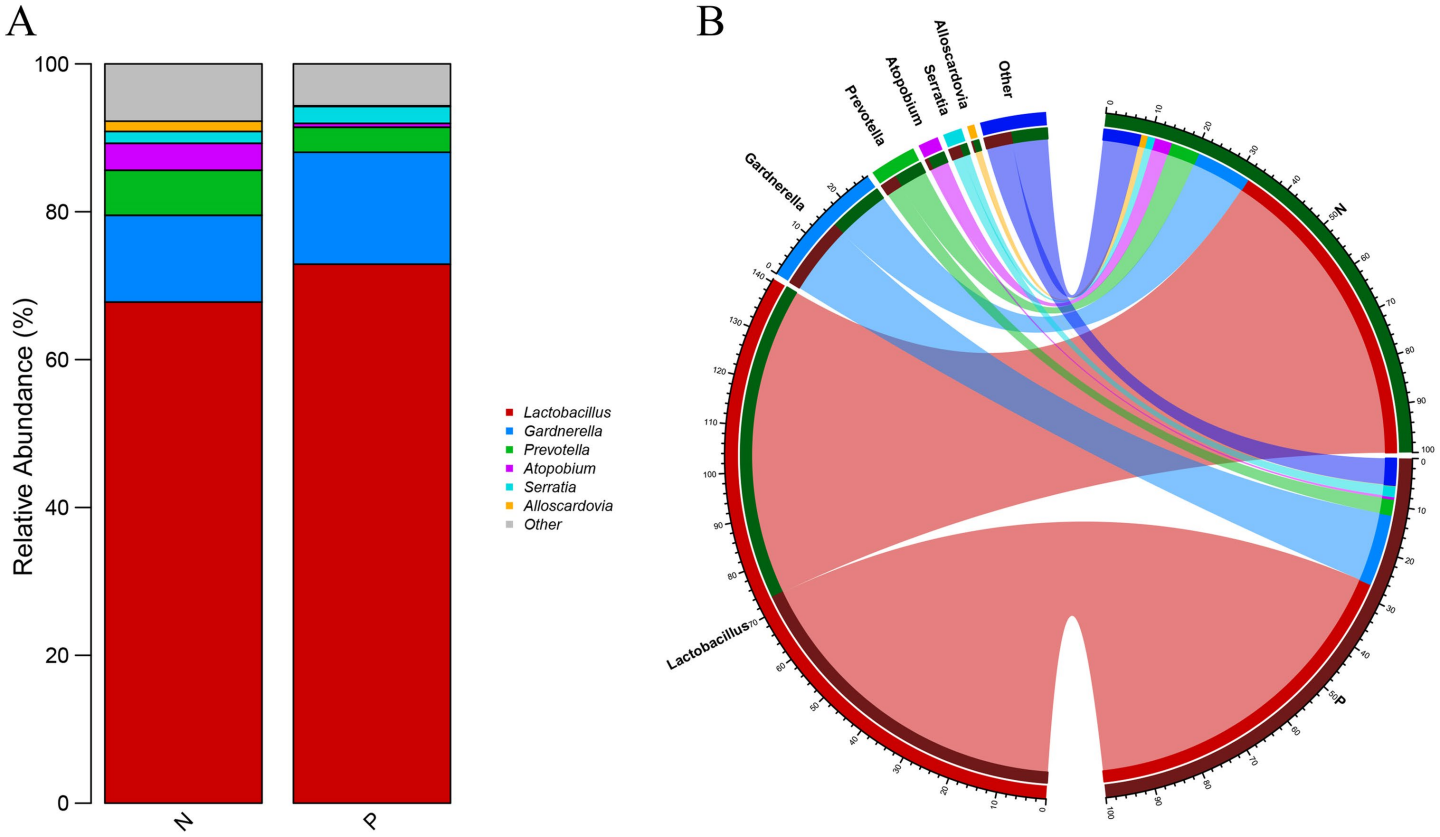
Comparative analysis of vaginal microbial abundance between pregnant and non-pregnant women. **(A)** Stacked bar plot showing genus-level relative abundance profiles; **(B)** circular plot illustrating the distribution (left semicircle) and group-specific abundance (right semicircle) of major genera.

To investigate potential differences in the diversity of the vaginal microbiota between pregnant (Group P) and non-pregnant (Group N) states, we compared the vaginal microorganisms of subjects in both groups. The relative abundance stacking plot (Figure 7A) and the covariance plot (Figure 7B) revealed that the percentage of *Lactobacillus* in Group P was higher than that in Group N. However, the diversity of the microbiota was lower in Group P compared to Group N (Kruskal–Wallis test with *p* < 0.05 and LDA score > 2.0). These findings suggest that the vaginal microbiota of women in the pregnant state is dominated by a higher proportion of *Lactobacillus*, indicating a more homogeneous diversity compared to the non-pregnant.

#### 3.5.3 Analysis of vaginal microbial diversity in women living in different regions

**Figure 8:**
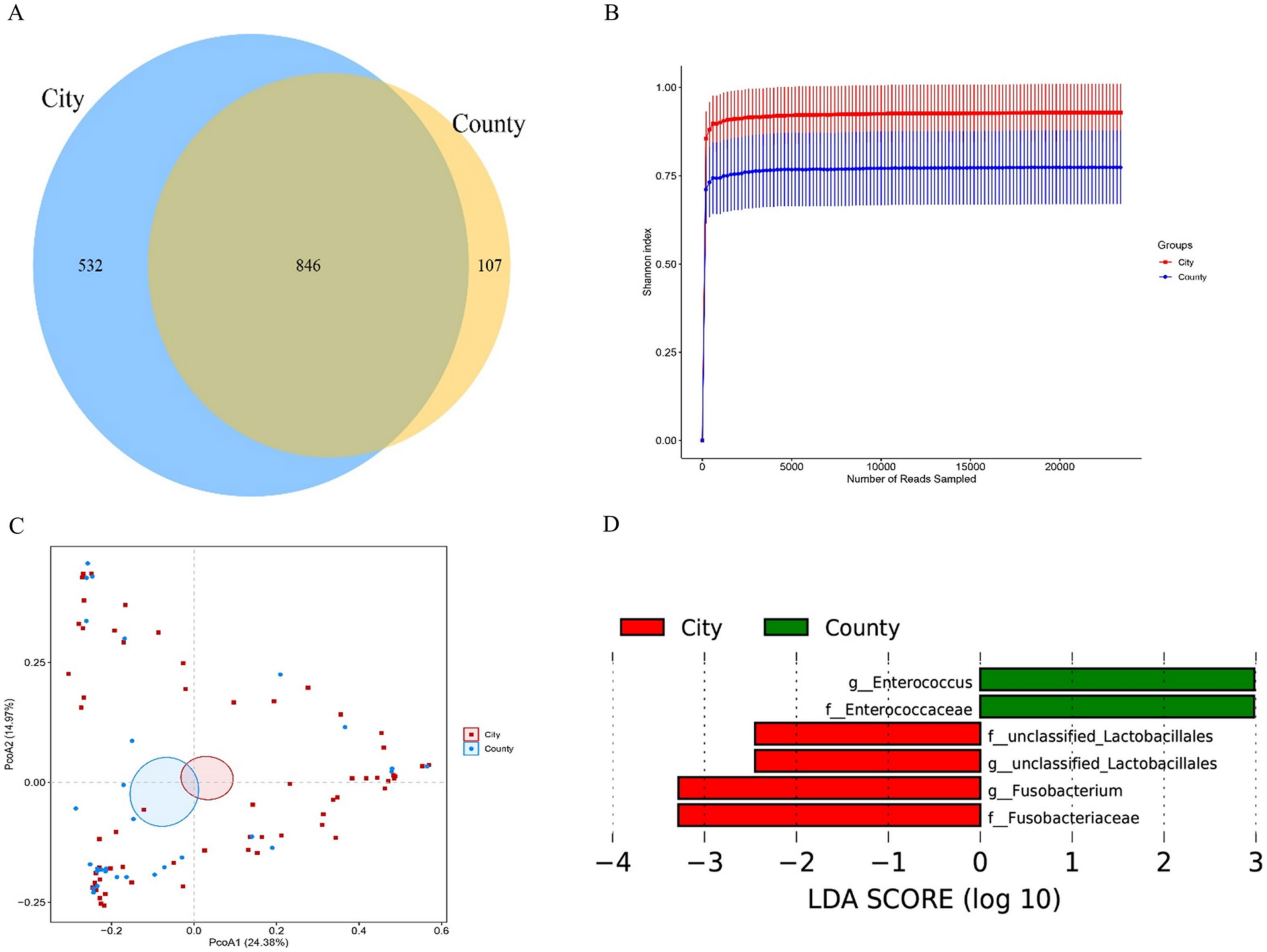
Vaginal microbiota composition across regional groups. **(A)** Venn diagram of shared and unique taxa; **(B)** Shannon’s exponential dilution graph; **(C)** PCoA analysis; **(D)** LEfSe analysis identifying differentially abundant taxa between groups.

To investigate potential differences in vaginal microbiota among women residing in different areas within the same urban, the samples were divided into urban groups and suburban groups based on their proximity to the urban center. Venn analysis (Figure 8A) was performed and data was shown in Table 2. The number of OTUs in the urban group was significantly higher than that in the suburban group (Kruskal–Wallis test with *p* < 0.05 and LDA score > 2.0), indicating that women in the urban group exhibited greater species richness in their vaginal flora compared to those in the suburban group. The rarefaction curves of samples from both the urban and suburban group (Figure 8B) demonstrated that the curves for the urban group were steeper than those for the suburban group. This indicates that the amount of sequenced microbial composition in the urban group was greater than in the suburban group, suggesting an increased vaginal microbial diversity in the urban group. Furthermore, the PCoA analysis (Figure 8C) showed a clear separation between the two groups, indicating the two groups could be distinguished well. Subsequently, the subgroup was subjected to LEfSe analysis to identify species that may significantly differ between the groups. The results (Figure 8D) revealed distinct microbial signatures between groups: *Enterococcaceae*, unclassified *Lactobacillales*, and *Fusobacterium* were enriched in the urban group. In contrast, the suburban group showed a significant increase in *Enterococcaceae* and its corresponding genus, *Enterococcus*. These findings suggest that the identified species may serve as microbial markers to distinguish between the two groups.

**Table 2:** OTU data of Venn profile in urban group and suburban group.

OTUs	Unique		Common	
	Quantity	Proportion	Quantity	Proportion
1,378	532	35.82%	846	56.97%
953	107	7.21%		
1,485				

OTU data of Venn profile in urban group and suburban group. Comparison of OTU numbers between groups was performed using independent samples t-test with FDR correction.

## 4 Discussion

This study utilized 16S rRNA gene sequencing to profile the vaginal microbiota of 100 healthy reproductive-age women. The primary objective was to determine whether this population could be categorized based on microbial composition. Based on operational taxonomic units (OTUs), cluster analysis enabled an initial classification into five major clusters and eight subtypes at the genus level. Ravel et al. (2011) first proposed a classification system for female vaginal communities, defining five community state types (CSTs). Among these, four are dominated by *Lactobacillus crispatus*, *Lactobacillus iners*, *Lactobacillus jensenii*, and *Lactobacillus gasseri* (CST I, II, III, and V, respectively), while the fifth group lacks *Lactobacillus* dominance (CST IV). Ravel’s classification was defined at the species level, whereas the first four classes identified in this study correspond to *Lactobacillus*-dominated CST types I, II, III, and V at the genus level. The fifth major group can be classified as the aforementioned non-*Lactobacillus* group (CST IV-A, IV-B). Overall, the findings of this study align with previous research in this field. Building on Ravel’s foundational work, subsequent studies have further explored various dimensions, including gynecological conditions, racial differences, and age-related changes in women. These studies consistently confirm the presence of the four common *Lactobacillus* species in healthy adult women, though their relative proportions vary depending on the specific research objectives and study populations. Consequently, these findings offer valuable insights for guiding clinical practice (Shuting and Rongxia, 2018) and suggest that *Lactobacillus* may serve as a candidate marker for vaginal secretion identification in forensic contexts, though species-level resolution is needed for further validation.

Analysis of the distinct subgroups revealed age-related variations in vaginal microbiota composition among reproductive-age women, following a discernible trend characterized by a gradual decline in the proportion of *Lactobacillus* and a concurrent increase in microbial diversity with age. This pattern suggests that age is associated with vaginal microbiota composition, consistent with previous findings (Auriemma et al., 2021). However, due to substantial inter-individual variability within each age group, these trends alone are insufficient for individual-level predictions. The vaginal microbiota of pregnant women displayed increased stability and homogeneity in terms of species diversity, with *Lactobacillus* persisting as the dominant genus. This observation aligns with findings from previous studies (Romero et al., 2014). This shift is attributed to elevated levels of estrogen and progesterone during pregnancy, which promote the predominance of *Lactobacillus* species, thereby supporting fetal development and maintaining maternal homeostasis. Moreover, studies conducted in urban and suburban areas of the same urban have shown that the vaginal microbiota is more diverse in urban populations. This difference may be linked to more complex lifestyle factors (Joseph et al., 2021; Fan et al., 2024), though further research is needed to establish causal relationships.

Consistent with previous studies across different populations (Ravel et al., 2011), our findings confirm the dominance of *Lactobacillus* in healthy women (Benschop et al., 2012; Giampaoli et al., 2012). However, due to the genus-level resolution of our data, we were unable to assess species-level variations associated with ethnicity, which warrants further investigation using higher-resolution methods. Racial differences are reviewed and summarized in Supplementary Table S2. The dominance of *Lactobacillus* in vaginal samples suggests its potential utility as a marker for vaginal secretion identification, which may assist in determining the nature of biological evidence in cases such as sexual assault (Wohlfahrt et al., 2023). Ghemrawi et al. (2021) reported that the penile microbiota is distinct from that of the vagina, dominated by genera such as *Actinomyces*, *Staphylococcus*, and *Corynebacterium*, while pubic hair microbiota also exhibits sex-specific differences. Although the present study focused exclusively on female samples, these findings underscore the need for future comparative studies involving male samples to further evaluate the forensic utility of microbiota-based sex estimation.

Nevertheless, certain limitations should be acknowledged. First, the sample size is relatively small and restricted to a specific population in a limited geographic area. To improve the microbial database and enhance its practical value, it is recommended that future studies include larger cohorts covering multiple regions and diverse ethnic groups. Such expansion would enable comparisons of vaginal microbiota across different geographical settings, between racial populations, and within specific ethnic communities. Remote comparisons of samples from suspects or victims could also offer valuable forensic insights. Second, the selection of hypervariable target regions requires careful consideration. Although the 16S rRNA gene is widely used as a cost-effective marker for bacterial studies, sequencing different hypervariable regions can lead to variations in observed microbial diversity and composition (Ying et al., 2019). Third, the potential influence of sampling site within the vagina should be taken into account. Previous studies (Chen et al., 2017; Huang et al., 2015) have suggested that microbial community composition may differ between sites such as the posterior fornix and the mid-vaginal wall, though these differences were not statistically significant-highlighting the need for further investigation. Fourth, as a cross-sectional study, this research provides only a snapshot of microbial composition at a single time point. Longitudinal studies with dynamic follow-up are necessary to fully understand temporal changes in an individual’s vaginal microbiota. Fifth, there is currently no universally standardized protocol for vaginal microbiome studies. The health criteria applied in this study are consistent with those used in similar research and are based on clinical diagnoses of the enrolled participants.

Despite these limitations, as microbial databases expand, it may eventually become feasible to explore whether demographic characteristics can be inferred from microbial profiles. Such capabilities could, in the future, contribute to sample screening in forensic investigations.

## 5 Conclusion

In conclusion, this study employed 16S rRNA high-throughput sequencing to characterize the vaginal microbiota in 100 healthy reproductive-age women, confirming *Lactobacillus* as the dominant genus and revealing five major community types at the genus level. While associations with age, pregnancy, and geographic region were observed, the genus-level resolution and sample size limit direct forensic application. The confirmation of *Lactobacillus* as a core component supports its potential as a marker for vaginal fluid identification. Future research should focus on higher-resolution longitudinal studies with diverse populations to assess the temporal stability and inter-individual uniqueness of the vaginal microbiota, which is essential for determining its ultimate utility in forensic individualization.

## Data Availability

The data presented in the study are deposited in the NCBI BioProject repository, accession number PRJNA1449061. https://www.ncbi.nlm.nih.gov/bioproject/PRJNA1449061.

## References

[ref1] Allwood J. S. Fierer N. Dunn R. R. (2020). The future of environmental DNA in forensic science. Appl. Environ. Microbiol. 86, e01504–e01519. doi: 10.1128/AEM.01504-19, 31704676 PMC6952231

[ref2] Auriemma R. S. Scairati R. Del Vecchio G. Liccardi A. Verde N. Pirchio R. . (2021). The vaginal microbiome: a long urogenital colonization throughout woman life. Front. Cell. Infect. Microbiol. 11:686167. doi: 10.3389/fcimb.2021.686167, 34295836 PMC8290858

[ref3] Benschop C. C. Quaak F. C. Boon M. E. Sijen T. Kuiper I. (2012). Vaginal microbial flora analysis by next generation sequencing and microarrays; can microbes indicate vaginal origin in a forensic context? Int. J. Legal Med. 126, 303–310. doi: 10.1007/s00414-011-0660-8, 22282153

[ref4] Bing-Bing X. (2017). Study of molecular biology applied in vaginal microecology. Chin. J. Pract. Gynecol. Obstet. 33, 795–800. doi: 10.19538/j.fk2017080107

[ref5] Børsting C. Morling N. (2015). Next generation sequencing and its applications in forensic genetics. Forensic Sci. Int. Genet. 18, 78–89. doi: 10.1016/j.fsigen.2015.02.002, 25704953

[ref6] Chen C. Song X. Wei W. Zhong H. Dai J. Lan Z. . (2017). The microbiota continuum along the female reproductive tract and its relation to uterine-related diseases. Nat. Commun. 8:875. doi: 10.1038/s41467-017-00901-0, 29042534 PMC5645390

[ref7] Chen X. Xu J. Wang H. Luo J. Wang Z. Chen G. . (2021). Profiling the differences of gut microbial structure between schizophrenia patients with and without violent behaviors based on 16S rRNA gene sequencing. Int. J. Legal Med. 135, 131–141. doi: 10.1007/s00414-020-02439-1, 33067643

[ref8] Fan Z. Han D. Fan X. Zeng Y. Zhao L. (2024). Analysis of the correlation between cervical HPV infection, cervical lesions and vaginal microecology. Front. Cell. Infect. Microbiol. 14:1405789. doi: 10.3389/fcimb.2024.1405789, 39220285 PMC11362039

[ref9] France M. Alizadeh M. Brown S. Ma B. Ravel J. (2022). Towards a deeper understanding of the vaginal microbiota. Nat. Microbiol. 7, 367–378. doi: 10.1038/s41564-022-01083-2, 35246662 PMC8910585

[ref10] García M. G. Pérez-Cárceles M. D. Osuna E. Legaz I. (2020). Impact of the human microbiome in forensic sciences: a systematic review. Appl. Environ. Microbiol. 86, e01451–e01420. doi: 10.1128/AEM.01451-20, 32887714 PMC7642070

[ref11] Ghemrawi M. Torres A. R. Duncan G. Colwell R. Dadlani M. McCord B. (2021). The genital microbiome and its potential for detecting sexual assault. Forensic Sci. Int. Genet. 51:102432. doi: 10.1016/j.fsigen.2020.102432, 33307384

[ref12] Giampaoli S. Alessandrini F. Frajese G. V. Guglielmi G. Tagliabracci A. Berti A. (2018). Environmental microbiology: perspectives for legal and occupational medicine. Leg. Med. 35, 34–43. doi: 10.1016/j.legalmed.2018.09.014, 30268689

[ref13] Giampaoli S. Berti A. Valeriani F. Gianfranceschi G. Piccolella A. Buggiotti L. . (2012). Molecular identification of vaginal fluid by microbial signature. Forensic Sci. Int. Genet. 6, 559–564. doi: 10.1016/j.fsigen.2012.01.005, 22364791

[ref14] Grice E. A. Segre J. A. (2012). The human microbiome: our second genome. Annu. Rev. Genomics Hum. Genet. 13, 151–170. doi: 10.1146/annurev-genom-090711-163814, 22703178 PMC3518434

[ref15] Huang Y. E. Wang Y. He Y. Ji Y. Wang L. P. Sheng H. F. . (2015). Homogeneity of the vaginal microbiome at the cervix, posterior fornix, and vaginal canal in pregnant Chinese women. Microb. Ecol. 69, 407–414. doi: 10.1007/s00248-014-0487-1, 25230887

[ref16] Huang H. Yao T. Wu W. Zhai C. Guan T. Song Y. . (2019). Specific microbes of saliva and vaginal fluid of Guangdong Han females based on 16S rDNA high-throughput sequencing. Int. J. Legal Med. 133, 699–710. doi: 10.1007/s00414-018-1986-2, 30610448

[ref17] Joseph R. J. Ser H. L. Kuai Y. H. Tan L. T. Arasoo V. J. T. Letchumanan V. . (2021). Finding a balance in the vaginal microbiome: How do we treat and prevent the occurrence of bacterial vaginosis? Antibiotics 10:719. doi: 10.3390/antibiotics10060719, 34203908 PMC8232816

[ref18] Liang X. Han X. Liu C. Du W. Zhong P. Huang L. . (2022). Integrating the salivary microbiome in the forensic toolkit by 16S rRNA gene: potential application in body fluid identification and biogeographic inference. Int. J. Legal Med. 136, 975–985. doi: 10.1007/s00414-022-02831-z, 35536322

[ref20] Metcalf J. L. Xu Z. Z. Bouslimani A. Dorrestein P. Carter D. O. Knight R. (2017). Microbiome tools for forensic science. Trends Biotechnol. 35, 814–823. doi: 10.1016/j.tibtech.2017.03.006, 28366290

[ref21] Ravel J. Gajer P. Abdo Z. Schneider G. M. Koenig S. S. McCulle S. L. . (2011). Vaginal microbiome of reproductive-age women. Proc. Natl. Acad. Sci. USA 108, 4680–4687. doi: 10.1073/pnas.1002611107, 20534435 PMC3063603

[ref22] Romero R. Hassan S. S. Gajer P. Tarca A. L. Fadrosh D. W. Nikita L. . (2014). The composition and stability of the vaginal microbiota of normal pregnant women is different from that of non-pregnant women. Microbiome 2:4. doi: 10.1186/2049-2618-2-4, 24484853 PMC3916806

[ref23] Sa R. N. Cai L. Y. Wu H. J. Yan J. W. Liu X. Hu R. (2017). Application of metagenomics in forensic identification. J. Forensic Med. 33, 397–400. doi: 10.3969/j.issn.1004-5619.2017.04.014 29219273

[ref24] Schmedes S. E. Woerner A. Budowle B. (2020). “Forensic human identification using skin microbiome genetic signatures,” in Microbial Forensics, (San Diego: Academic Press).

[ref25] Schmedes S. E. Woerner A. E. Novroski N. M. M. Wendt F. R. King J. L. Stephens K. M. . (2018). Targeted sequencing of clade-specific markers from skin microbiomes for forensic human identification. Forensic Sci. Int.: Genet. 32, 50–61. doi: 10.1016/j.fsigen.2017.10.004, 29065388

[ref26] Shuting W. Rongxia H. E. (2018). Progress in research on metagenomics of vaginal microecosystem. Chin. J. Microecol. 30, 114–120. doi: 10.13381/j.cnki.cjm.201801028

[ref27] Turnbaugh P. J. Ley R. E. Hamady M. Fraser-Liggett C. M. Knight R. Gordon J. I. (2007). The human microbiome project. Nature 449, 804–810. doi: 10.1038/nature06244, 17943116 PMC3709439

[ref28] Wohlfahrt D. Tan-Torres A. L. Green R. Brim K. Bradley N. Brand A. . (2023). A bacterial signature-based method for the identification of seven forensically relevant human body fluids. Forensic Sci. Int. Genet. 65:102865. doi: 10.1016/j.fsigen.2023.102865, 37004371

[ref29] Yao T. Wang Z. Liang X. Liu C. Yu Z. Han X. . (2021). Signatures of vaginal microbiota by 16S rRNA gene: potential bio-geographical application in Chinese Han from three regions of China. Int. J. Legal Med. 135, 1213–1224. doi: 10.1007/s00414-021-02525-y, 33594458

[ref19] Ying L. Qiongqiong Z. Lei Z. Ying W. Tao L. Zhi T. . (2019). Comparison of different 16S rDNA hypervariable regions selection for the microbiome study of bacterial vaginosis. Prog. Obstet. Gynecol. 28, 804–807. doi: 10.13283/j.cnki.xdfckjz.2019.11.003

