# Supplementary material for: Characterization of vaginal microbiota diversity by 16S rRNA high-throughput sequencing
Source: Front Microbiol. 2026 Apr 20;17:1777216. doi: 10.3389/fmicb.2026.1777216 (PMC13138041; doi:10.3389/fmicb.2026.1777216)
Supplement: Supplementary file 1 [file Table_1.docx]

**Supplementary table 1** Details of the sample

| Sample number | Lablel | Age group | Pregnancy states | Region | Cleanliness | Trichomonas | H_2_O_2_ | Leukocyte esterase | PH (≤4.6) | Lactobacillus | BV | Candida |
| --- | --- | --- | --- | --- | --- | --- | --- | --- | --- | --- | --- | --- |
| 1 | 101505 | 40-50 | Non-pregnancy | Urban | Ⅱ | — | — | — | √ | much | — | — |
| 2 | 101508 | 20-29 | pregnancy | Urban | Ⅱ | — | — | ＋ | √ | much | — | — |
| 3 | 101603 | 30-39 | pregnancy | Urban | Ⅲ | — | ＋ | ＋ | √ | little | — | — |
| 4 | 101604 | 20-29 | Non-pregnancy | Urban | Ⅱ | — | ＋ | ＋ | √ | little | — | — |
| 5 | 101610 | 40-50 | pregnancy | Urban | Ⅲ | — | ＋ | ＋ | √ | little | — | — |
| 6 | 101611 | 30-39 | pregnancy | Urban | Ⅱ | — | ＋ | ＋ | √ | medium | — | — |
| 7 | 101614 | 20-29 | Non-pregnancy | Urban | Ⅲ | — | ＋ | ＋ | √ | little | — | — |
| 8 | 101617 | 20-29 | Non-pregnancy | Urban | Ⅱ | — | — | ＋ | √ | much | — | — |
| 9 | 101619 | 30-39 | Non-pregnancy | Suburban | Ⅱ | — | ＋ | ＋ | √ | little | — | — |
| 10 | 101724 | 20-29 | Non-pregnancy | Urban | Ⅲ | — | — | ＋ | √ | much | — | — |
| 11 | 101725 | 20-29 | pregnancy | Urban | Ⅱ | — | ＋ | ＋ | √ | medium | — | — |
| 12 | 101726 | 30-39 | Non-pregnancy | Suburban | Ⅲ | — | ＋ | ＋ | √ | little | — | — |
| 13 | 101801 | 30-39 | Non-pregnancy | Urban | Ⅱ | — | — | ＋ | √ | much | — | — |
| 14 | 101802 | 20-29 | Non-pregnancy | Urban | Ⅲ | — | ＋ | ＋ | √ | little | — | — |
| 15 | 101803 | 30-39 | pregnancy | Suburban | Ⅳ | — | ＋ | ＋ | √ | medium | ＋ | — |
| 16 | 101804 | 20-29 | pregnancy | Urban | Ⅲ | — | ＋ | ＋ | √ | little | — | — |
| 17 | 101806 | 30-39 | Non-pregnancy | Suburban | Ⅱ | — | — | ＋ | √ | much | — | — |
| 18 | 101901 | 30-39 | pregnancy | Urban | Ⅲ | — | ＋ | ＋ | √ | little | — | — |
| 19 | 102101 | 30-39 | Non-pregnancy | Suburban | Ⅲ | — | ＋ | ＋ | √ | little | — | — |
| 20 | 102103 | 30-39 | pregnancy | Suburban | Ⅲ | — | ＋ | ＋ | √ | little | — | — |
| 21 | 102217 | 20-29 | pregnancy | Urban | Ⅲ | — | ＋ | ＋ | √ | little | — | — |
| 22 | 102305 | 40-50 | Non-pregnancy | Suburban | Ⅱ | — | — | ＋ | √ | much | — | — |
| 23 | 102309 | 20-29 | pregnancy | Suburban | Ⅱ | — | — | ＋ | √ | much | — | — |
| 24 | 102403 | 30-39 | pregnancy | Urban | Ⅱ | — | ＋ | ＋ | √ | medium | — | — |
| 25 | 102502 | 40-50 | Non-pregnancy | Urban | Ⅰ | — | — | — | √ | much | — | — |
| 26 | 102503 | 30-39 | pregnancy | Urban | Ⅱ | — | ＋ | — | √ | little | — | — |
| 27 | 102504 | 30-39 | pregnancy | Urban | Ⅱ | — | ＋ | — | √ | little | — | — |
| 28 | 102505 | 30-39 | pregnancy | Urban | Ⅱ | — | ＋ | — | √ | little | — | — |
| 29 | 102506 | 20-29 | Non-pregnancy | Urban | Ⅲ | — | ＋ | ＋ | √ | little | — | — |
| 30 | 102507 | 30-39 | pregnancy | Urban | Ⅱ | — | ＋ | — | √ | little | — | — |
| 31 | 102601 | 20-29 | pregnancy | Suburban | Ⅱ | — | — | ＋ | √ | much | — | — |
| 32 | 102603 | 30-39 | Non-pregnancy | Suburban | Ⅱ | — | — | ＋ | √ | much | — | — |
| 33 | 102604 | 20-29 | Non-pregnancy | Urban | Ⅱ | — | ＋ | ＋ | √ | medium | — | — |
| 34 | 102804 | 30-39 | pregnancy | Urban | Ⅱ | — | ＋ | ＋ | √ | medium | — | — |
| 35 | 102805 | 20-29 | Non-pregnancy | Suburban | Ⅱ | — | ＋ | ＋ | √ | medium | — | — |
| 36 | 102806 | 30-39 | Non-pregnancy | Urban | Ⅱ | — | ＋ | ＋ | √ | medium | — | — |
| 37 | 102807 | 20-29 | pregnancy | Urban | Ⅱ | — | ＋ | ＋ | √ | medium | — | — |
| 38 | 102809 | 20-29 | Non-pregnancy | Urban | Ⅲ | — | ＋ | ＋ | √ | little | — | — |
| 39 | 102810 | 20-29 | pregnancy | Urban | Ⅲ | — | ＋ | ＋ | √ | little | — | — |
| 40 | 102812 | 30-39 | pregnancy | Suburban | Ⅱ | — | — | ＋ | √ | much | — | — |
| 41 | 102907 | 30-39 | Non-pregnancy | Suburban | Ⅱ | — | — | ＋ | √ | much | — | — |
| 42 | 103002 | 30-39 | pregnancy | Urban | Ⅲ | — | ＋ | ＋ | √ | little | — | — |
| 43 | 103004 | 30-39 | Non-pregnancy | Urban | Ⅱ | — | — | ＋ | √ | much | — | — |
| 44 | 103102 | 20-29 | pregnancy | Urban | Ⅲ | — | ＋ | ＋ | √ | little | — | — |
| 45 | 103103 | 30-39 | pregnancy | Urban | Ⅱ | — | — | ＋ | √ | much | — | — |
| 46 | 110108 | 20-29 | Non-pregnancy | Suburban | Ⅲ | — | — | ＋ | √ | much | — | — |
| 47 | 110109 | 30-39 | Non-pregnancy | Urban | Ⅱ | — | — | ＋ | √ | much | — | — |
| 48 | 110112 | 30-39 | pregnancy | Suburban | Ⅱ | — | — | ＋ | √ | much | — | — |
| 49 | 110202 | 30-39 | pregnancy | Suburban | Ⅱ | — | — | ＋ | √ | much | — | — |
| 50 | 110204 | 20-29 | Non-pregnancy | Urban | Ⅱ | — | — | ＋ | √ | much | — | — |
| 51 | 110205 | 30-39 | pregnancy | Urban | Ⅲ | — | ＋ | ＋ | √ | little | — | — |
| 52 | 110301 | 20-29 | pregnancy | Urban | Ⅲ | — | ＋ | ＋ | √ | little | — | — |
| 53 | 110304 | 40-50 | pregnancy | Urban | Ⅱ | — | — | ＋ | √ | much | — | — |
| 54 | 110306 | 20-29 | Non-pregnancy | Urban | Ⅲ | — | ＋ | ＋ | √ | little | — | — |
| 55 | 110401 | 30-39 | Non-pregnancy | Urban | Ⅱ | — | ＋ | ＋ | √ | medium | — | — |
| 56 | 110502 | 30-39 | Non-pregnancy | Suburban | Ⅱ | — | — | ＋ | √ | much | — | — |
| 57 | 110503 | 30-39 | pregnancy | Urban | Ⅱ | — | — | ＋ | √ | much | — | — |
| 58 | 110504 | 30-39 | pregnancy | Urban | Ⅱ | — | ＋ | ＋ | √ | medium | — | — |
| 59 | 110505 | 20-29 | pregnancy | Urban | Ⅱ | — | ＋ | ＋ | √ | medium | — | — |
| 60 | 110506 | 40-50 | Non-pregnancy | Suburban | Ⅱ | — | — | ＋ | √ | much | — | — |
| 61 | 110507 | 20-29 | pregnancy | Urban | Ⅱ | — | — | ＋ | √ | much | — | — |
| 62 | 110601 | 30-39 | Non-pregnancy | Suburban | Ⅱ | — | ＋ | ＋ | √ | little | — | — |
| 63 | 110602 | 30-39 | pregnancy | Urban | Ⅱ | — | ＋ | ＋ | √ | little | — | — |
| 64 | 110612 | 20-29 | Non-pregnancy | Suburban | Ⅲ | — | ＋ | ＋ | √ | medium | — | — |
| 65 | 110615 | 30-39 | pregnancy | Urban | Ⅲ | — | ＋ | ＋ | √ | little | — | — |
| 66 | 110616 | 20-29 | Non-pregnancy | Suburban | Ⅱ | — | — | ＋ | √ | much | — | — |
| 67 | 110617 | 30-39 | pregnancy | Suburban | Ⅱ | — | — | ＋ | √ | much | — | — |
| 68 | 110901 | 20-29 | Non-pregnancy | Suburban | Ⅱ | — | — | ＋ | √ | much | — | — |
| 69 | 111002 | 30-39 | pregnancy | Suburban | Ⅱ | — | — | ＋ | √ | much | — | — |
| 70 | 111003 | 20-29 | Non-pregnancy | Suburban | Ⅲ | — | ＋ | ＋ | √ | little | — | — |
| 71 | 111102 | 30-39 | pregnancy | Urban | Ⅲ | — | ＋ | ＋ | √ | little | — | — |
| 72 | 111103 | 20-29 | Non-pregnancy | Urban | Ⅱ | — | — | ＋ | √ | much | — | — |
| 73 | 111208 | 30-39 | Non-pregnancy | Suburban | Ⅲ | — | ＋ | ＋ | √ | little | — | — |
| 74 | 111213 | 40-50 | Non-pregnancy | Urban | Ⅱ | — | ＋ | ＋ | √ | medium | — | — |
| 75 | 111506 | 30-39 | Non-pregnancy | Suburban | Ⅲ | — | ＋ | ＋ | √ | little | — | — |
| 76 | 111508 | 20-29 | pregnancy | Urban | Ⅲ | — | ＋ | ＋ | √ | little | — | — |
| 77 | 111510 | 30-39 | pregnancy | Suburban | Ⅲ | — | ＋ | ＋ | √ | little | — | — |
| 78 | 111804 | 30-39 | pregnancy | Urban | Ⅱ | — | — | ＋ | √ | medium | — | — |
| 79 | 111805 | 40-50 | Non-pregnancy | Urban | Ⅱ | — | ＋ | ＋ | √ | medium | — | — |
| 80 | 111806 | 40-50 | Non-pregnancy | Urban | Ⅱ | — | — | ＋ | √ | medium | — | — |
| 81 | 111808 | 20-29 | pregnancy | Urban | Ⅲ | — | ＋ | ＋ | √ | medium | — | — |
| 82 | 111809 | 30-39 | pregnancy | Urban | Ⅲ | — | ＋ | ＋ | √ | little | — | — |
| 83 | 112001 | 40-50 | Non-pregnancy | Suburban | Ⅱ | — | ＋ | ＋ | √ | medium | — | — |
| 84 | 112101 | 30-39 | pregnancy | Suburban | Ⅲ | — | ＋ | ＋ | √ | little | — | — |
| 85 | 112102 | 40-50 | Non-pregnancy | Suburban | Ⅲ | — | ＋ | ＋ | √ | little |  |  |
| 86 | 113001 | 30-39 | pregnancy | Suburban | Ⅱ | — | ＋ | ＋ | √ | medium | — | — |
| 87 | 121001 | 30-39 | pregnancy | Suburban | Ⅱ | — | — | ＋ | √ | much | — | — |
| 88 | 121007 | 30-39 | Non-pregnancy | Suburban | Ⅱ | — | ＋ | ＋ | √ | medium | — | — |
| 89 | 121806 | 20-29 | Non-pregnancy | Suburban | Ⅱ | — | ＋ | ＋ | √ | medium | — | — |
| 90 | 122201 | 30-39 | pregnancy | Suburban | Ⅲ | — | ＋ | ＋ | √ | little | — | — |
| 91 | 122202 | 20-29 | Non-pregnancy | Suburban | Ⅲ | — | ＋ | ＋ | √ | little | — | — |
| 92 | 122203 | 30-39 | pregnancy | Suburban | Ⅱ | — | ＋ | ＋ | √ | medium | — | — |
| 93 | 122204 | 30-39 | pregnancy | Suburban | Ⅲ | — | ＋ | ＋ | √ | little | — | — |
| 94 | 122701 | 30-39 | pregnancy | Suburban | Ⅱ | — | — | ＋ | √ | much | — | — |
| 95 | 122702 | 30-39 | pregnancy | Suburban | Ⅱ | — | — | ＋ | √ | much | — | — |
| 96 | 122703 | 20-29 | pregnancy | Suburban | Ⅱ | — | — | ＋ | √ | much | — | — |
| 97 | 010201 | 40-50 | Non-pregnancy | Suburban | Ⅱ | — | — | ＋ | √ | much | — | — |
| 98 | 010203 | 40-50 | Non-pregnancy | Suburban | Ⅲ | — | ＋ | ＋ | √ | little | — | — |
| 99 | 010204 | 40-50 | Non-pregnancy | Suburban | Ⅲ | — | ＋ | ＋ | √ | little | — | — |
| 100 | 010501 | 40-50 | Non-pregnancy | Suburban | Ⅱ | — | — | ＋ | √ | much | — | — |

Note: ”—” means negative; ”＋” means positive; “√” means meeting the condition.
