# Supplementary material for: Characterization of vaginal microbiota diversity by 16S rRNA high-throughput sequencing
Source: Front Microbiol. 2026 Apr 20;17:1777216. doi: 10.3389/fmicb.2026.1777216 (PMC13138041; doi:10.3389/fmicb.2026.1777216)
Supplement: Supplementary file 2 [file Table_2.docx]

|  | Phylum | Genus | Spices | Data source | | | |
| --- | --- | --- | --- | --- | --- | --- | --- |
|  |  |  |  | Research team | Sample source | Method | Sample size |
| Asian | Firmicutes, Actinobacteri, Bacteroidetes, Proteobacteria, Fusobacteria, Tenericutes | *Lactobacillus, Gardnerella, Prevotella, Serratia, Atopobium, Bifidobacterium, Anaerococcus, Streptococcus* | *Lactobacillus crispatus, Lactobacillus iners, Lactobacillus jensenii, Lactobacillus gasseri* | \| Shi (1) \| Chinese \| 16S rRNA \| 5 \| \| --- \| --- \| --- \| --- \| \| Lee (2) \| Korean \| 16S rRNA \| 136 \| \| Zhou(3) \| Japanese \| 16S rRNA \| 73 \| \| Zhou(4) \| Caucasians \| 16S rRNA \| 5 \| \| Li (5) \| Chinese \| whole macrogenome \| 52 \| \| Zhou (6) \| North America (Caucasians, black female) \| 16S rRNA \| 144 \| \| Ravel (7) \| Caucasians, black female, Hispanics, Asian \| 16S rRNA \| 396 \| \| Liu (8) \| Chinese \| whole macrogenome \| 215 \| \| Kinsley (9) \| Nigerian \| other \| 241 \| \| Jeniffer (10) \| African- American, European \| 16S rRNA \| 1684 \| \| Noyes (11) \| Hispanics, Caucasians, black female, Asian \| 16S rRNA \| 396 \| | | | |
| Caucasians |  | *Lactobacillus, Megasphaera, Atopobium, Leptotrichia,* | *Lactobacillus jensenii, Lactobacillus crispatus* |  |  |  |  |
| European |  |  | *Lactobacillus jensenii, Lactobacillus gasseri, Lactobacillus crispatus* |  |  |  |  |
| American |  |  | *Lactobacillus jensenii* |  |  |  |  |
| African |  | *Megasphaera, Micrococcus, Anaerobicoccus, Fingonella, Peptostreptococcus* | *Multi-colony dominant state under non-lactobacillus dominance* |  |  |  |  |
| Hispanics |  | *Lactobacillus, Gardnerella, Unclassified Bacteria, Eggerthella* |  |  |  |  |  |
| African- American |  |  |  |  |  |  |  |

**Supplementary Table S2** Summary of vaginal microbiology study among women in different races.

1. Shi Y, Chen L, Tong J, Xu C. Preliminary characterization of vaginal microbiota in healthy Chinese women using cultivation-independent methods. The journal of obstetrics and gynaecology research. 2009;35(3):525-32.

2. Lee JE, Lee S, Lee H, Song YM, Lee K, Han MJ, et al. Association of the vaginal microbiota with human papillomavirus infection in a Korean twin cohort. PloS one. 2013;8(5):e63514.

3. Zhou X, Hansmann MA, Davis CC, Suzuki H, Brown CJ, Schütte U, et al. The vaginal bacterial communities of Japanese women resemble those of women in other racial groups. FEMS immunology and medical microbiology. 2010;58(2):169-81.

4. Zhou X, Brown CJ, Abdo Z, Davis CC, Hansmann MA, Joyce P, et al. Differences in the composition of vaginal microbial communities found in healthy Caucasian and black women. The ISME journal. 2007;1(2):121-33.

5. Li F, Chen C, Wei W, Wang Z, Dai J, Hao L, et al. The metagenome of the female upper reproductive tract. GigaScience. 2018;7(10).

6. Zhou X, Bent SJ, Schneider MG, Davis CC, Islam MR, Forney LJ. Characterization of vaginal microbial communities in adult healthy women using cultivation-independent methods. Microbiology (Reading, England). 2004;150(Pt 8):2565-73.

7. Ravel J, Gajer P, Abdo Z, Schneider GM, Koenig SS, McCulle SL, et al. Vaginal microbiome of reproductive-age women. Proceedings of the National Academy of Sciences of the United States of America. 2011;108 Suppl 1(Suppl 1):4680-7.

8. Liu F, Zhou Y, Zhu L, Wang Z, Ma L, He Y, et al. Comparative metagenomic analysis of the vaginal microbiome in healthy women. Synthetic and systems biotechnology. 2021;6(2):77-84.

9. Anukam KC, Osazuwa EO, Ahonkhai I, Reid G. Lactobacillus vaginal microbiota of women attending a reproductive health care service in Benin city, Nigeria. Sexually transmitted diseases. 2006;33(1):59-62.

10. Fettweis JM, Brooks JP, Serrano MG, Sheth NU, Girerd PH, Edwards DJ, et al. Differences in vaginal microbiome in African American women versus women of European ancestry. Microbiology (Reading, England). 2014;160(Pt 10):2272-82.

11. Noyes N, Cho KC, Ravel J, Forney LJ, Abdo Z. Associations between sexual habits, menstrual hygiene practices, demographics and the vaginal microbiome as revealed by Bayesian network analysis. PloS one. 2018;13(1):e0191625.
